# Functionally Convergent B Cell Receptor Sequences in Transgenic Rats Expressing a Human B Cell Repertoire in Response to Tetanus Toxoid and Measles Antigens

**DOI:** 10.3389/fimmu.2017.01834

**Published:** 2017-12-22

**Authors:** Jean-Philippe Bürckert, Axel R. S. X. Dubois, William J. Faison, Sophie Farinelle, Emilie Charpentier, Regina Sinner, Anke Wienecke-Baldacchino, Claude P. Muller

**Affiliations:** ^1^Department of Infection and Immunity, Luxembourg Institute of Health, Esch-sur-Alzette, Luxembourg

**Keywords:** transgenic rats, B cell repertoire, next-generation sequencing, repertoire convergence, public CDR3s, AIRR-seq, DESeq2

## Abstract

The identification and tracking of antigen-specific immunoglobulin (Ig) sequences within total Ig repertoires is central to high-throughput sequencing (HTS) studies of infections or vaccinations. In this context, public Ig sequences shared by different individuals exposed to the same antigen could be valuable markers for tracing back infections, measuring vaccine immunogenicity, and perhaps ultimately allow the reconstruction of the immunological history of an individual. Here, we immunized groups of transgenic rats expressing human Ig against tetanus toxoid (TT), Modified Vaccinia virus Ankara (MVA), measles virus hemagglutinin and fusion proteins expressed on MVA, and the environmental carcinogen benzo[a]pyrene, coupled to TT. We showed that these antigens impose a selective pressure causing the Ig heavy chain (IgH) repertoires of the rats to converge toward the expression of antibodies with highly similar IgH CDR3 amino acid sequences. We present a computational approach, similar to differential gene expression analysis, that selects for clusters of CDR3s with 80% similarity, significantly overrepresented within the different groups of immunized rats. These IgH clusters represent antigen-induced IgH signatures exhibiting stereotypic amino acid patterns including previously described TT- and measles-specific IgH sequences. Our data suggest that with the presented methodology, transgenic Ig rats can be utilized as a model to identify antigen-induced, human IgH signatures to a variety of different antigens.

## Introduction

Immunoglobulin (Ig) molecules are the primary effectors of the humoral immune response. In theory, Ig can bind to every possible antigen through the large variety of Ig V (variable), D (diversity), and J (joining) gene rearrangements in the bone marrow (BM) and target-oriented affinity maturation in germinal centers (1). All B cells of a germinal center are clonally related to a common ancestor and target the same antigen with varying affinities, iteratively selecting for improved affinity and avidity (2). The Ig molecules of the emerging B cells bind to the target epitope in a lock-and-key principle which is mediated mainly by the heavy chain complementary-determining region 3 (CDR3) loop on top of the Ig (1, 3). The CDR3 is the most variable part of the Ig sequence and the main antigen-binding determinant. The repertoire of CDR3s sufficiently describes the entire functional Ig heavy chain (IgH) repertoire of an individual (3, 4).

High-throughput sequencing (HTS) has been widely applied to study the IgH repertoire in response to vaccination and infection (5). With this technique, it has become possible to investigate the evolutionary affinity maturation processes after antigenic challenge and to compare their outcome across individuals (6). The IgH repertoire is essentially private (7), but it appears that individuals also produce a public response to a common antigenic stimulus characterized by a certain degree of similarity at the CDR3 sequence level (8–11). Public CDR3s were notably identified in human in response to dengue infection, H1N1 seasonal influenza vaccination, and repetitive polysaccharide antigens (5, 11, 12). Such CDR3s provided signatures of past immunological exposures allowing for sequence-based monitoring of vaccination or infectious diseases, and perhaps ultimately to reconstruct an individual’s antigenic history. Studies investigating this concept of public Ig CDR3s mainly used human blood-derived PBMCs. These represent only a miniscule part of the complete Ig repertoire (13), and it is critical to capture the affinity-matured B cells during their brief transit from the germinal centers through peripheral blood to the BM. The large heterogeneity of human B cell repertoires composed of past exposure to a plethora of antigens further complicates the identification of antigen-induced Ig sequences in the context of single antigen challenge or vaccination (14). The usage of an animal model provides ready access to secondary lymphoid organs after restricted antigen exposure, enabling a focused investigation of antigen-experienced plasma cells (15–17).

Here, we applied HTS on class switched, BM B cells, rich in serum antibody producing plasma cells, from rats carrying human germline IgH and light chain (IgL) loci, the OmniRat™ (18–21). These transgenic rats were immunized with viral (Modified Vaccinia virus Ankara, MVA), protein [measles virus (MV) hemagglutinin and fusion proteins, HF, and tetanus toxoid (TT)], and chemically defined hapten–conjugate antigens (benzo[a]pyrene-TT, BaP-TT) to study the evolution of convergent CDR3 amino acid sequences. We showed that OmniRat™ mount convergent Ig responses characterized by CDR3s with high amino acid sequence similarity. The level of similarity was consistent for all investigated antigens. We applied an approach similar to differential expression analysis to identify overrepresented clusters of highly similar, antigen-driven CDR3s. These could be grouped into antigen-associated signatures matching previously described MV-specific OmniRat™ hybridomas (22) and human TT-specific antibodies (12, 23–25). Our results suggested that humanized Ig transgenic rats can be used as a model to study human-like Ig repertoire dynamics and to determine antigen-associated CDR3 signatures to characterize the history of antigen exposure in human individuals.

## Materials and Methods

### Animals and Immunizations

Humanized Ig transgenic rats (OmniRat™, Open Monoclonal Technology Inc., Palo Alto, CA, USA) were developed and bred as previously described (18–21). OmniRat™ carry a chimeric human/rat Ig heavy chain locus, where 22 human IgHV genes and all human IgHD and IgHJ genes are linked to the rat IgHC genes in germline configuration as well as fully human, Ig light chain lambda, and kappa loci (20) (Table S1 in Supplementary Material). A total of 32 animals were separated into six groups of four to six individuals. They received three intraperitoneal injections at 2-week intervals and were sacrificed 7 days after the last injection. Injections either contained 100 µg of TT (TT group, *n* = 4; Serum Institute of India, Pune, India) or of a BaP-TT conjugate construct (BaP-TT group, *n* = 5) (26), both formulated with 330 µg of aluminum hydroxide (ALUM). Other rats were injected with 10^7^ PFU of a recombinant MVA expressing the hemagglutinin (H) and fusion (F) glycoproteins of the MV (MVA-HF group, *n* = 6) or the MVA viral vector only (MVA group, *n* = 6) without adjuvant. The control animals received either 330 µg of ALUM alone (ALUM group, *n* = 6) or were left untouched (NEG group, *n* = 5). Antigen-specific IgG responses were monitored by ELISA 10 days after immunizations and at sacrifice. All animal procedures were in compliance with the rules described in the Guide for the Care and Use of Laboratory Animals (27) and accepted by the “Comité National d’Éthique de Recherche” (Luxembourg).

### Antigens for Immunization and ELISA

BaP was coupled to ovalbumin (OVA, Sigma-Aldrich) for ELISA and to purified TT as previously described (26). The recombinant MVA and the recombinant MVA carrying MV H and F proteins of the Edmonston strain [MV vaccine strain, clade A] viruses were propagated on BHK-21 cells (ATTC™ CCL-10™) as previously described (28–30). Antigen-specific IgG antibody levels in sera were determined in 384-well microtiter plates (Greiner bio-one, Wemmel, Belgium), coated overnight at 4°C with either 250 ng of MV antigen (Measles grade 2 antigens; Microbix Biosystems, Mississauga, ON, USA), 2.5 × 10^5^ PFU of sonicated MVA (~314 ng), 187.5 ng of TT, or 0.25 µM of BaP-OVA in carbonate buffer (100 mM, pH 9.6). Free binding sites were saturated with 1% bovine serum albumin in Tris-buffered saline at room temperature for 2 h. Serial dilutions of the sera were added for 90 min at 37°C and developed with alkaline phosphatase-conjugated goat anti-rat IgG (1/750 dilution; ImTec Diagnostics, Antwerp, Belgium) and the appropriate substrate. Absorbance was measured at 405 nm. End point titers were determined as the serum dilutions corresponding to five times the background.

### Sample Preparation, Amplification, and IonTorrent PGM Sequencing

Lymphocytes were isolated from BM samples by density-gradient centrifugation (ficoll^®^ Paque Plus; Sigma-Aldrich). Total RNA was extracted from 10^8^ cells with an RNeasy midi kit following the manufacturer’s protocol (Qiagen) and enriched for mRNA using paramagnetic separation (μMACS mRNA Isolation kit; Miltenyi Biotec, Leiden, Netherlands). cDNA was prepared from 300 ng of mRNA using dT_18_ primers and Superscript III reverse transcriptase (Thermo Fisher Scientific) at 50°C for 80 min. Recombined IgH fragments were subsequently amplified by multiplex PCR using primers for human IgHV region and rat Cγ region with Q5 Hot Start High Fidelity polymerase (NEB, Ipswich, MA, USA) as described previously (22). Amplicons were size selected on a 2% agarose gel and quantified. Quality was checked with a Bioanalyzer (High Sensitivity DNA, Agilent Technologies, Diegem, Belgium). Four randomly selected libraries were pooled in equimolar concentrations and sequenced on a 318™ Chip v2 (Thermo Fisher Scientific) using multiplex identifiers (MIDs) with the Ion OneTouch™ Template OT2 400 Kit and the Ion PGM Sequencing 400 Kit (Thermo Fisher Scientific) on the Ion Torrent Ion Personal Genome Machine (PGM™) System (Thermo Fischer Scientific).

### Quality Control and Sequence Annotation

BAM files were extracted from the Torrent Suite^TM^ software (version 4.0.2, standard settings) and demultiplexed by MIDs. Only reads with an unambiguously assigned MID (0 mismatch), identified primers at both ends (two mismatches allowed) and more than 85% of the bases with a quality score above 25 were considered for further analysis. After clipping MIDs and primers, sequences were collapsed and submitted to the ImMunoGeneTics database (IMGT) HighV-QUEST web server^1^ (31) for IgHV gene annotation and CDR3 delineation (32). IgHV and IgHJ genes for the inframe, productive sequences were subsequently assigned using a local installation of IgBlast (33), including only the genes present in the genome of the OmniRat™ as references. Only sequences with an unambiguously assigned IgHV and IgHJ gene were considered for further analysis.

Human Ig sequences (IgG and IgM) were obtained from the Sequence Read Archive database^2^ and processed as described [Accession number: SRP068407 (34)]. Samples taken at day 7 post immunization were excluded to avoid skewing of data distributions by the applied vaccination. Samples were annotated using IMGT and postprocessed using Change-O framework [v 0.2.4 (35)]. Only functional sequences present at least three times per data set were considered for assessing CDR3 length distributions and somatic hypermutation level.

### CDR3 Similarity Threshold for Public Immune Responses

The number of matches for the 200 most frequent CDR3s (top 200) of a rat A in a rat B was obtained for a series of similarity thresholds and returned as ratio from 0 to 1 (i.e., all top 200 CDR3s of rat A have a match in rat B). Ratios were determined from 50–100% sequence similarity in 1% increments. The averages for the top 200 matching ratios at each increment were then calculated for all rats within a vaccination group and all rats vaccinated with unrelated antigens. Rats with related antigens were excluded in the pairwise comparison (e.g., MVA as intragroup for MVA-HF). The average top 200 matching ratios were plotted against sequence similarity along with the first derivatives in GraphPad^3^ Prism 5.

### Identification of Antigen-Driven CDR3 Clusters

Only CDR3s longer than four amino acids were included in the analysis. CDR3s with a minimum of 80% amino acid similarity were considered as relatives. One amino acid length difference was allowed, to account for insertion and deletions introduced by SHM and occasional differential CDR3-IgHJ region alignments by IMGT (36, 37). Length difference was penalized the same way as a substitution. For each CDR3, the cumulative count of all its 80% relatives per rat (CDR3 count) was calculated and stored in a fuzzy match count table. Data were imported and analyzed with DESeq2 according to the standard workflow for RNA-seq, treating CDR3 counts as expression values (38). Briefly, data were imported as a count-data matrix and converted into a DESeq2-object with conditions according to the antigens used for vaccination. Correct sample grouping was confirmed using variance stabilizing transformation count data (VST counts). Euclidian distance computation was performed on VST counts as described in the DESeq2 vignette (39). Principle component analysis plots were generated using the “PlotPCA” function on VST counts of the DESeq2 package. *P*-values were adjusted for multiple testing and to determine the false discovery rate (FDR) using Benjamini–Hochberg correction (39). Based on an FDR of 1%, overrepresented CDR3 sequences were extracted if their adjusted *P*-values were lower than 0.01. Log2-fold change cutoffs were determined manually per antigen group. The extracted CDR3 sequences were grouped using single-seed iterative clustering based on maximum difference of 80% sequence similarity. All analytical scripts were written in Python 2.7 and R 3.2.3 (40).

### 3D Modeling

Selected Ig nucleotide sequences were uploaded to IMGT for annotation. Sequences were elongated to full length by adding the missing nucleotides from the closest germline gene as predicted by the IMGT algorithm. Full length sequences were submitted to the “Rosetta Online Server that Includes Everyone” [ROSIE^4^ (41–43)], with enabled H3 loop modeling option. ROSIE-output PDB files of the grafted and relaxed models were visualized using PyMol^5^ [version 1.7.4 (44)].

## Results

### High-Throughput Sequencing of OmniRat™ IgH mRNA Transcripts

To study convergent IgH repertoires in response to vaccination, 32 transgenic Ig humanized rats (OmniRat™) were immunized with different antigens (Table 1; TT, BaP-TT, MVA, and MVA-HF). ALUM was used as an adjuvant for TT and BaP-TT. Two control groups received either the adjuvant alone or were left untouched (NEG). All animals exhibited a specific antibody response against the immunizations and mock immunized (ALUM group) and non-immunized animals (NEG group) showed no detectable antigen-specific antibodies (Figure S1 in Supplementary Material). MVA-HF- and BaP-TT-vaccinated animals exhibited a specific immune response against the MVA vector or the TT carrier protein, respectively, albeit at lower levels than the animals immunized with these antigens only (Figure S1 in Supplementary Material). Rearranged heavy chain IgG genes were amplified from mRNA extracted from BM lymphocytes and sequenced on a HTS Ion Torrent PGM™ platform. A total of 37,473,982 raw reads with MID were obtained (range: 850,298–1,879,372 per animal, Table S2 in Supplementary Material). After quality control and annotation, on average 86,619 unique nt sequences per animal were retained for analysis. The rats expressed a diverse IgH repertoire, including varying frequencies of all human IgHV and IgHJ genes. All possible IgHVJ combinations were found in all vaccination groups with no bias in IgHV, IgHJ genes, or IgHVJ recombination usage. The CDR3 length distribution of rats was comparable to that of observed in human IgG B cells (34) and somatic hypermutation resulted in an average germline similarity of 97.27% (±1.9%, Figure S2 in Supplementary Material).

**Table 1:** Study design: antigen and vaccination groups.

Antigen	TT group		MVA group		Controls	
	TT (4)	BaP-TT (5)	MVA (6)	MVA-HF (6)	ALUM (6)	NEG (5)
Benzo[a]pyrene	–	x	–	–	–	–
TT	x	(x)	–	–	–	–
Measles virus H + F	–	–	–	x	–	–
MVA	–	–	x	x	–	–
Alum	x	x	–	–	x	–

*Group sizes are indicated in parentheses. TT used in the BaP-TT vaccination group was chemically modified during the coupling process and therefore does not present the same antigenic surface as native TT in the TT group*.

### Unique and Highly Similar CDR3 Sequences in Response to the Same Antigen

**Figure 1:**
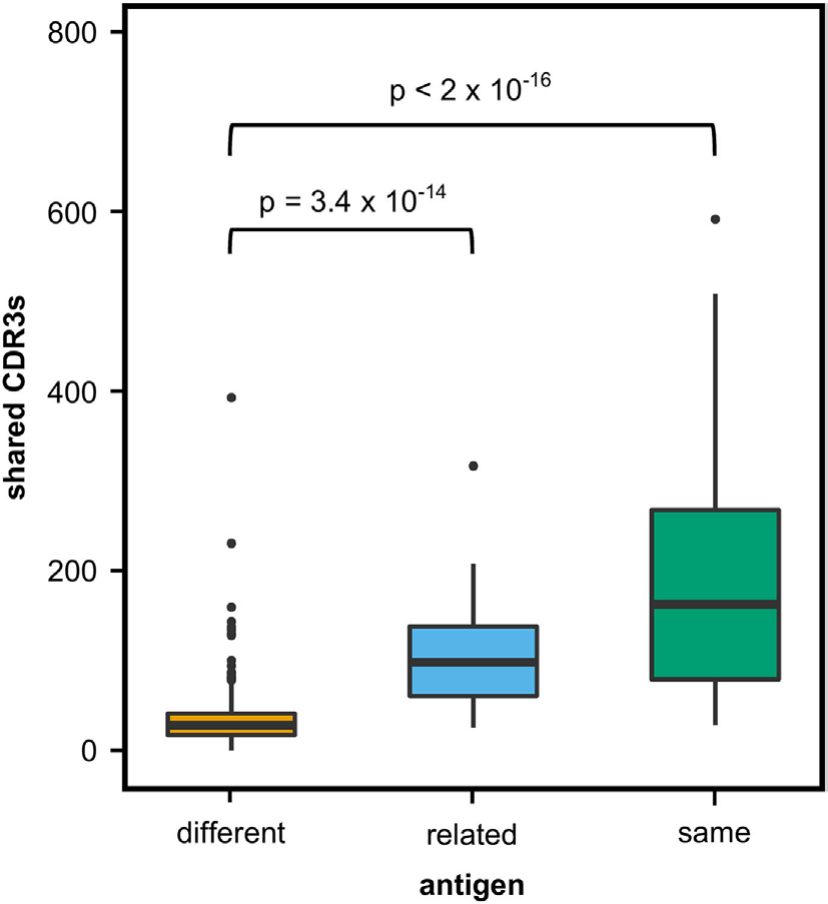
Shared complementary-determining region 3 (CDR3s) in OmniRat™ pairs. Box–whisker plots represent the number of identical CDR3s shared between pairs of rats from different (369 pairs, orange), related (56 pairs, light blue), or the same vaccination group (71 pairs, green). More CDR3s were shared between rats from the same (*P*-value 3.4 × 10^−14^) or related (*P*-value < 2 × 10^−16^) antigen group than between rats of different antigen groups (Kruskal–Wallis test followed by Nemenyi *post hoc* test).

We first investigated to what extent rats that received the same antigen expressed the same CDR3 amino acid sequences. Pairs of rats from different vaccination groups (369 pairs) shared less CDR3s with each other than pairs of rats within the same group (71 pairs, *P*-value < 2 × 10^−16^, Kruskal–Wallis with Nemenyi *post hoc* test) or immunized with related antigens (56 pairs, *P*-value = 3.4 × 10^−14^), indicating that mutual CDR3s are essentially induced by the immunizations (Figure 1). Among a total of 11,643 identical CDR3s (i.e., 100% similarity) that were shared by any set of two or more rats irrespective of the antigen, 5,346 CDR3s (45.9%) were shared exclusively by animals of the same group and 1,912 (16.4%) were shared between animals immunized with a related antigen (TT and BaP-TT, MVA, and MVA-HF). Most of the CDR3s shared within groups were common to only two animals of the same group (6,467; 89.1% of CDR3s shared within groups only). CDR3s present in all animals of a group were rare (Table 2). For instance, only a single CDR3 was shared between all 6 rats immunized with MVA-HF, and 3 CDR3s were shared between all the 12 animals exposed to the MVA vector (combined MVA and MVA-HF group) (Table 2). However, multiple CDR3s, differing only by one or two amino acids, were shared by all animals within a vaccination group but not by animals from other groups (Table 3). Interestingly, these differences occurred preferentially at certain positions of a CDR3 amino acid sequence. This suggested that the vaccinations seemed to have induced identical CDR3s as well as clusters of highly similar CDR3s.

**Table 2:** Number of identical complementary-determining region 3 (CDR3s) shared between rats in the same vaccination group or immunized with related antigens.

Group	Not shared	Number of CDR3s shared by *n* animals per group										
		*n* = 2	3	4	5	6	7	8	9	10	11	12
BaP-TT	27,167	510	74	12	0	–	–	–	–	–	–	–
TT	27,317	988	75	15	–	–	–	–	–	–	–	–
MVA	61,139	1,123	168	40	1	0	–	–	–	–	–	–
MVA-HF	43,778	686	51	4	0	1	–	–	–	–	–	–
NEG	33,675	455	37	3	0	–	–	–	–	–	–	–
BaP-TT + TT	/	258	101	24	11	0	1	1	0	–	–	–
MVA + MVA-HF	/	924	316	102	72	35	29	15	8	8	4	3

**Table 3:** Alignment of selected complementary-determining region 3 (CDR3) (≥80% similarity) shared by rats in the Modified Vaccinia virus Ankara (MVA)-HF group highlighting the amino acid variation across animals being predominant at certain positions of the CDR3.

MVA-HF-associated CDR3s																	No. of animals
A	R	I	V	G	A	T	T	E	F	D	Y						6
–	–	–	–	–	–	–	–	D	–	–	–						4
–	–	V	–	–	–	–	–	–	–	–	–						4
–	–	A	–	–	–	–	–	–	–	–	–						3
–	–	–	–	–	–	S	–	D	–	–	–						2
–	–	V	–	–	–	–	–	D	–	–	–						2
–	–	–	–	–	–	S	–	–	–	–	S						2
–	–	A	–	–	–	S	–	–	–	–	–						2
–	–	–	–	–	–	S	–	–	–	–	C						2
–	–	–	–	–	–	S	N	–	–	–	–						2
–	–	–	–	–	D	S	–	–	–	–	–						2
–	–	G	–	–	–	–	–	–	–	–	–						2
A	R	H	R	T	Y	Y	Y	G	S	G	S	P	L	F	D	Y	4
–	–	–	–	–	F	–	–	–	–	–	–	–	–	–	–	–	4
–	–	–	–	–	–	–	–	–	–	–	–	–	P	–	–	–	3
–	–	–	–	–	–	–	–	–	–	–	–	–	H	–	–	–	2
–	–	–	–	–	H	–	–	–	–	–	–	–	–	–	–	–	2
–	–	–	–	–	–	–	F	–	–	–	–	–	–	–	–	–	2
–	–	–	Q	–	–	–	–	–	–	–	–	–	R	–	–	P	2
–	–	–	–	–	F	–	F	–	–	–	–	–	–	–	–	–	2
–	–	–	–	–	H	–	–	–	–	–	–	–	I	–	–	–	2
–	–	–	–	–	–	–	–	–	–	–	–	–	–	–	–	P	2
–	–	–	–	–	F	–	F	–	–	–	–	–	R	–	–	P	2
–	–	–	K	–	F	–	–	–	–	–	–	–	R	–	–	–	2
–	–	–	–	–	–	–	–	–	–	–	–	–	I	–	–	–	2
–	–	–	–	–	–	–	–	–	–	–	–	–	R	–	–	–	2

### Shared Antigen-Related CDR3s at 80% Sequence Similarity

**Figure 2:**
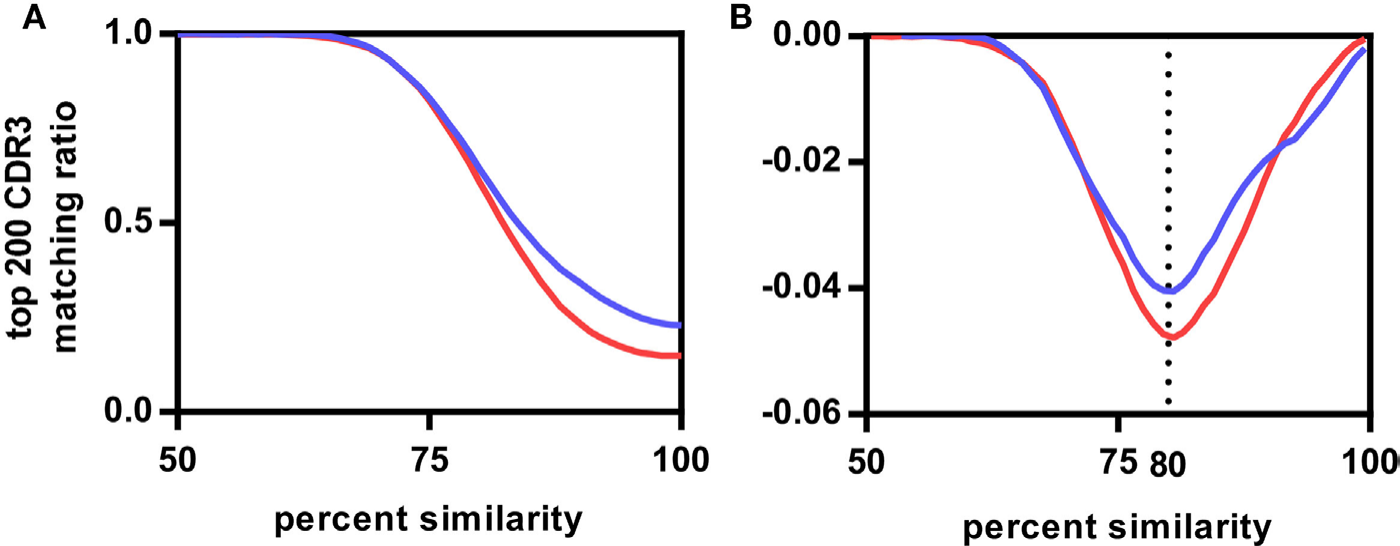
Influence of complementary-determining region 3 (CDR3) sequence similarity on CDR3 repertoire overlap between rats. **(A)** Average fractions of top 200 CDR3s of the Modified Vaccinia virus Ankara (MVA)-HF vaccination group shared with all CDR3s of other samples. Samples were divided into two groups having either the same antigen (MVA-HF group samples, blue curve) or different antigens (aluminum hydroxide, benzo[a]pyrene-tetanus toxoid, tetanus toxoid, and NEG samples, red curve), samples from overlapping antigens (in this case the MVA group) were excluded from this. Both curves follow a similar sigmoidal behavior. **(B)** First derivative of both curves. Inflection points align at 80% CDR3 amino acid similarity.

We compared CDR3s within and across the different vaccination groups to estimate the degree of similarity between these antigen-related clusters. We determined which of the top 200 CDR3s, representing on average 72.1 ± 7.6% of the repertoire of the rats (Figure S3 in Supplementary Material), of any rat A had a related CDR3 in a rat B either within the same group (intragroup comparison) or between groups (intergroup comparison) allowing for a single amino acid substitution. The same analysis was repeated for two, three, and up to eight amino acid substitutions. The number of top 200 CDR3s found to be present in inter- and intragroup was plotted against the amino acid substitutions expressed as percentage of CDR3 length (Figure 2A; Figure S4 in Supplementary Material). The resulting sigmoidal curves showed a similar shape for all vaccination groups. In the exponential phase between 100% and 90–95%, intragroup overlap was higher than intergroup overlap. In the linear phase between 90–95% and 75%, overlap increased faster for the intergroup comparison. In the asymptotic phase below 75% similarity, both inter- and intragroup overlap leveled off toward 1, indicating that all top 200 CDR3s of a rat had relatives in any other rat, irrespective of the antigen administered. The first derivatives of the curves showed that in all cases the inflection point was at around 80% (Figure 2B; Figure S4 in Supplementary Material). Thus, at this similarity threshold a maximum number of related CDR3s can be found within the same group while keeping the number of related CDR3s between groups at a minimum. In conclusion, all antigens induced in these rats a public IgH response that can best be characterized by clusters of CDR3s with at least 80% similarity.

### Hierarchical Clustering of CDR3 Repertoires at 80% Sequence Similarity

**Figure 3:**
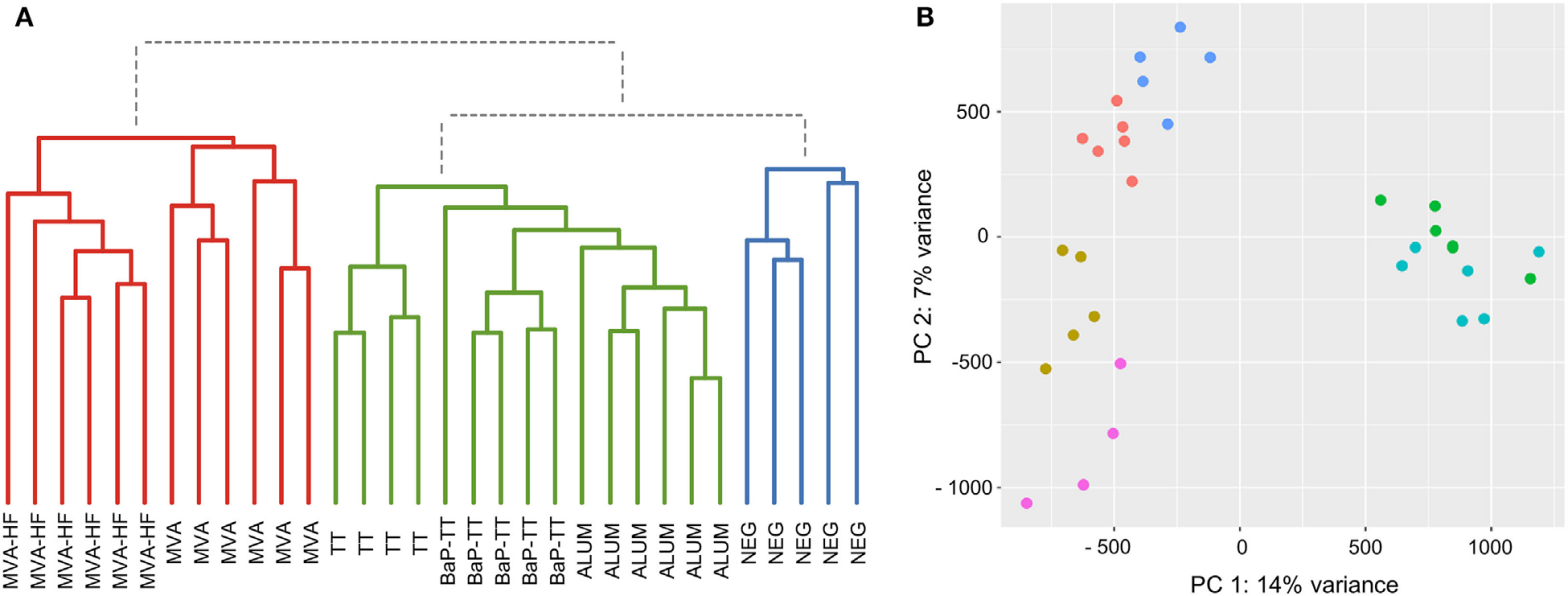
Sample grouping for 80% complementary-determining region 3 similarity counts. **(A)** Dendrogram of the Euclidian sample distances calculated for variance stabilizing transformation (VST) counts. Three main clusters are indicated by coloration (Cluster I: red, Cluster II: green, and Cluster III: blue). **(B)** Scatter plot for the first two principal components of VST counts. Samples are colored by vaccination-group [Modified Vaccinia virus Ankara (MVA): light blue, MVA-HF: green, tetanus toxoid: pink, benzo[a]pyrene-tetanus toxoid: gold, aluminum hydroxide: red, and NEG: blue].

Based on the above observation, we identified antigen-driven CDR3s using a workflow developed for differential gene expression analysis of RNA-seq data (38). For each CDR3 within a rat, counts of CDR3 sequences with 80% similarity (CDR3 counts) were used analogous to RNA-seq read counts. Rats of the same vaccination group were considered as replicates. The CDR3 counts followed a negative binomial distribution (Figure S5A in Supplementary Material). Compared to RNA-seq data, CDR3s usually lack a baseline expression and are essentially private, resulting mostly in zero counts for individuals across the study, while some shared CDR3s have very high counts in a single animal (Figure S5B in Supplementary Material). To account for this distribution, we applied VST to the CDR3 counts reducing the variance of the SDs over ranked mean values (Figure S5C in Supplementary Material). Hierarchical clustering of VST counts revealed three clusters (Figure 3A). Cluster I included all animals immunized with MVA (with or without MV HF protein expression). Interestingly, within this cluster, animals of the MVA-HF group and of the MVA group emerged from two separate branches indicating that additional presentation of HF antigens leaves a distinct imprint in the CDR3 repertoire. Cluster II contained the three groups of animals that received alum as an adjuvant (TT, BaP-TT, and ALUM). Again, each of the three groups clustered on separated sub-branches. Cluster III contained only untreated animals (NEG group) and was distinct from all immunized animals. The low variance and the specific grouping of the samples through both principle components showed that the VST counts cluster the data by the vaccination group. This indicated that the different antigens had distinct impact on a subset of the Ig repertoire of the rats (Figure 3B). When the data were reanalyzed applying an 85 or 75% threshold, the clear clustering of rats by vaccination group was lost (Figure S6 in Supplementary Material), thus confirming that the 80% similarity threshold was optimal to identify antigen-associated responses on the CDR3 repertoire of the rats. Additionally, it showed that VST CDR3 count data can be analyzed analogously to RNA-seq count data.

### Large Numbers of Antigen-Associated CDR3s Group into Stereotypic Signatures

Similar to RNA-seq expression experiments, we aimed to identify CDR3s that are differentially represented between groups of rats. Based on an FDR of 1%, 16,727 of the 249,657 (6.9%) unique CDR3s across all groups were found to be overrepresented. One hundred-fold differences in numbers of overrepresented CDR3s were identified in each of the six antigen groups (Table 4). The highest number of overrepresented CDR3s was found in the two combined groups MVA and MVA-HF (*n* = 11,080, 10.4% of the unique CDR3s for this combined group) and TT and BaP-TT (*n* = 2,451, 4.4%), which reflected the high immunogenicity of the antigens TT and MVA common within these groups. Less overrepresented CDR3s were found in the MVA (*n* = 1,689, 2.6%), the MVA-HF (*n* = 804, 1.8%), and TT group (*n* = 540, 1.8%). The lowest number of overrepresented CDR3s was found in the BaP-TT group (*n* = 163, 0.6%). These overrepresented CDR3s could be considered group-specific and thus immunization induced.

**Table 4:** Antigen-driven sequences and 80% similarity clusters.

Vaccination group	Antigen	Overexpressed complementary-determining region 3	No. of clusters (*n* > 10)	Total clusters
Benzo[a]pyrene-tetanus toxoid (BaP-TT)	Benzo[a]pyrene + tetanus toxoid (TT)	163	3	20
TT	TT	540	14	46
Modified Vaccinia virus Ankara (MVA)-HF	Measles virus H + F	804	13	79
MVA	MVA	1,689	28	99
BaP-TT and TT	TT backbone	2,451	25	63
MVA-HF and MVA	MVA backbone	11,080	233	419

**Figure 4:**
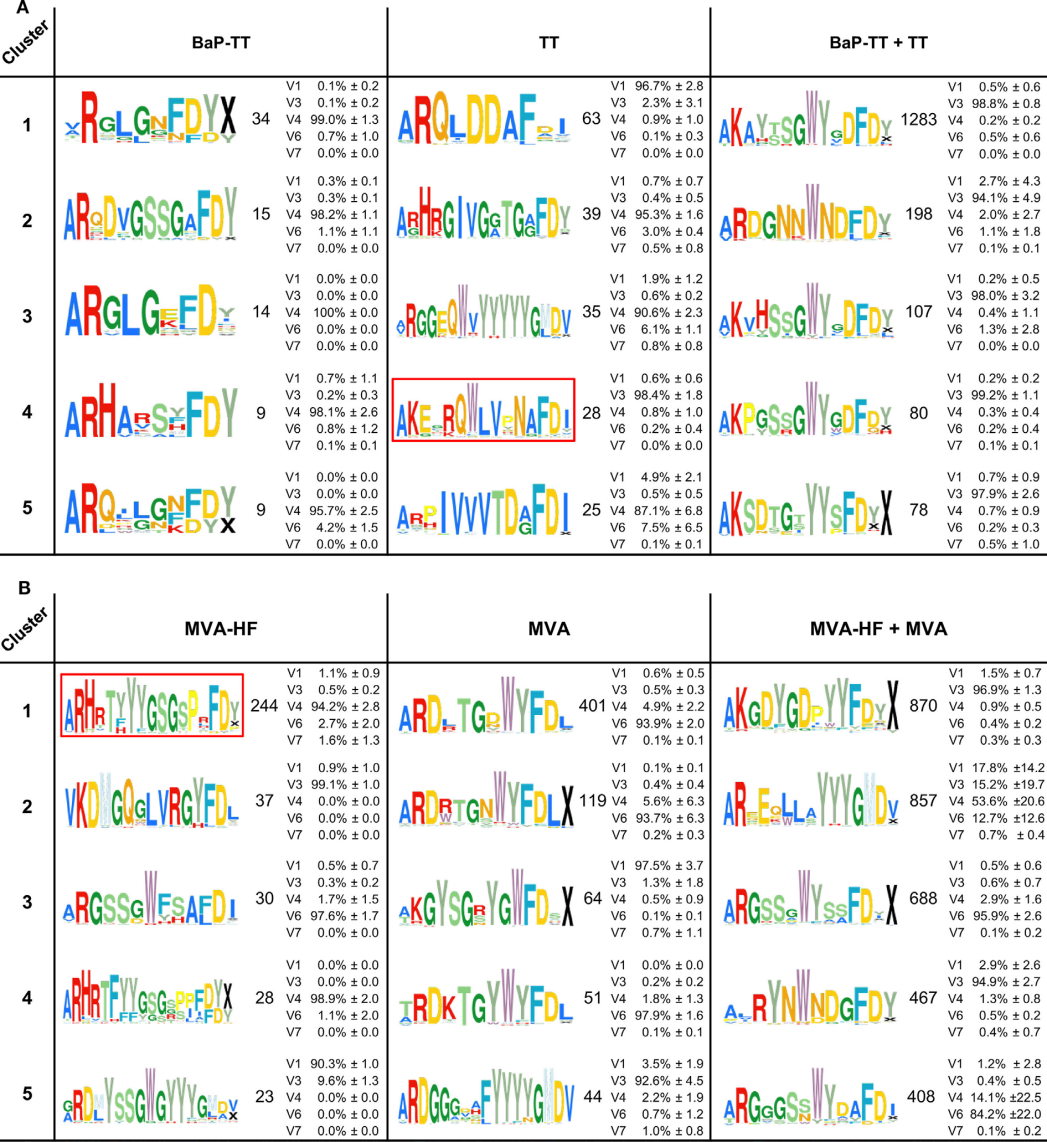
Antigen-associated complementary-determining region 3 (CDR3)-similarity clusters. The top five clusters of 80% similar CDR3s overexpressed in response to the antigens are shown as Weblogos. Coloration follows ImMunoGeneTics database amino acid coloration scheme (45). Numbers represent the unique CDR3s in each cluster. Average IgHV gene family usage (±SD) is indicated as percentage of sequences across all rats per cluster (see also Table S3 in Supplementary Material). IgHV2 was excluded, as no sequences in the clusters were derived from this family. **(A)** Clusters associated with the antigens benzo[a]pyrene-tetanus toxoid (BaP-TT), tetanus toxoid (TT), and the combined antigen groups BaP-TT and TT. The red box indicates TT-associated OmniRat™ CDR3s bearing an amino acid pattern also found in human anti-TT PBMC CDR3 sequences for independent studies. **(B)** Clusters associated with the antigens Modified Vaccinia virus Ankara (MVA)-HF, MVA, and the combined antigens MVA and MVA-HF. The red box indicates measles virus (MV)-HF associated CDR3 signature also identified in OmniRat™ hybridomas generated in an independent experiment in response to MV antigens.

**Figure 5:**
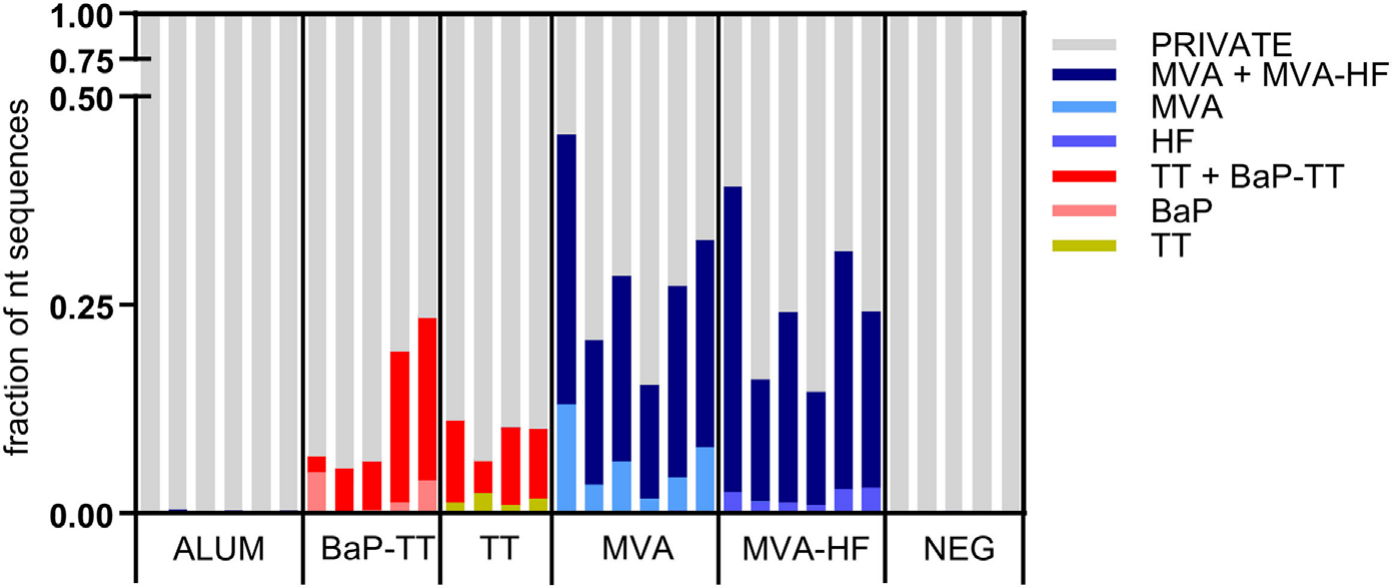
Fractions of the nucleotide immunoglobulin (Ig) repertoire encoding for complementary-determining region 3 (CDR3) signatures. The Ig repertoire per sample is displayed using numbers of full length nucleotide sequences. Nucleotide sequences encoding for CDR3s that are part of a signature are colored by associated antigen.

Overrepresented CDR3s were grouped into clusters of 80% sequence similarity (Figure 4). The larger the antigen, the more clusters were found. For instance, 20 clusters were found for the BaP-hapten while 109 clusters were found for the TT protein (46 for TT alone and 63 for TT and BaP-TT combined). The largest number of clusters was found for the MVA virus antigen (518, with 99 for MVA alone, and 419 for MVA and MVA-HF combined). These complex antigen-driven clusters of CDR3s, typical for each group, represented up to 46.5% of the BM IgH repertoire of the rats (Figure 5). The fraction of the repertoire corresponding to these CDR3 clusters varied between the groups but was relatively consistent among animals of the same antigen group.

Sequences encoding the overrepresented CDR3s were surprisingly diverse in IgHV gene usage (Table S3 in Supplementary Material). IgHV genes belonging to one family were largely predominant for each cluster (average 93.3 ± 9.8% of genes belonging to one family per cluster), and the level of associated IgHV genes was comparable between rats of each cluster (Figure 4; Table S3 in Supplementary Material). Interestingly, *cluster 2* of the combined group MVA + MVA-HF showed an elevated IgHV gene family repertoire. The dominant IgHV4 gene family accounted only for 57.5% (±20.4%) of the CDR3s, and the IgHV gene families IgVH1 (16.3 ± 13.4%), IgVH3 (14.2 ± 18.5%), and IgVH6 (11.3 ± 11.6%) were predominating the *cluster 2* repertoire of one, two, and one rat, respectively. All together we showed that OmniRat™ exhibited large fractions of highly similar, stereotypic CDR3s in response to the applied vaccinations, even across groups with shared antigens.

### Stereotypic Signatures Match Known MV-Specific and TT-Specific CDR3s

**Figure 6:**
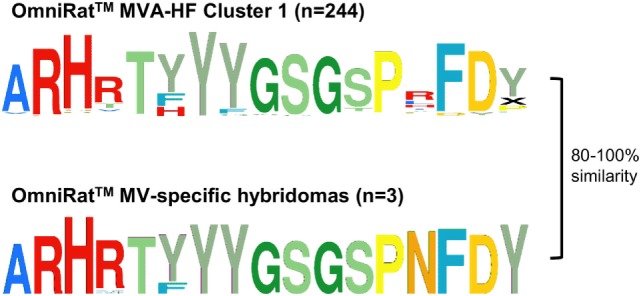
OmniRat™ measles virus (MV)-specific complementary-determining region 3 (CDR3) signature. The clusters of CDR3s overrepresented in response to Modified Vaccinia virus Ankara (MVA)-HF (see also Figure 4A), and the CDR3s from three monoclonal hybridomas specific for MV proteins are shown as Weblogos. The differences between the sequences were calculated as Levenshtein distances in percentage of CDR3 length.

**Figure 7:**
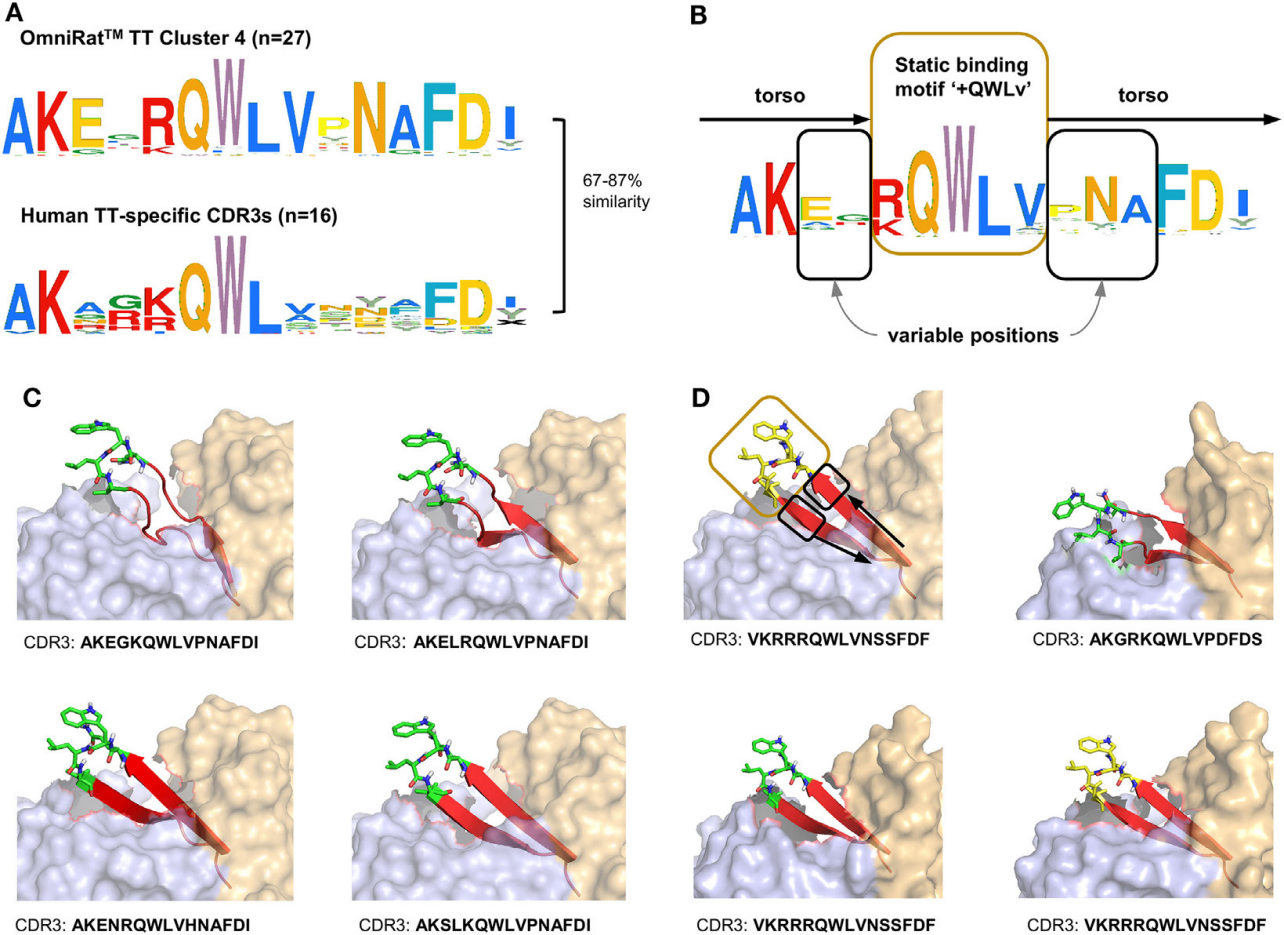
Omnirat™ and human antibodies against tetanus toxoid (TT) with similar properties and structures. **(A)** Sequence similarity range between OmniRat™ TT-associated *cluster 4* (Figure 4B) complementary-determining region 3 (CDR3s) and human TT-specific CDR3s (Levenshtein distance as percentage of sequence length). **(B)** Amino acid pattern for the combined TT-specific human and TT-associated rat CDR3s. The Weblogo shows the conserved binding motif “+QWLV” (+ indicates K/R), and torso amino acids with variable positions are highlighted. **(C)** 3D-homology models of four OmniRat™-HC-human-LC chimeric antibody Fab fragments. Heavy chains are colored in orange and light chains in blue, both visualized with 50% transparent surface. CDR3 torsos are shown as cartoon and colored in red. Binding motifs are displayed as sticks and colored in green with only polar hydrogens shown. Views were enlarged to focus on the CDR3 structure. **(D)** 3D-homology models of four human Fab fragments visualized as described for **(C)**. Motif, variable and torso structures are highlighted with boxes and arrows as described in **(B)**.

Recombinant Modified Vaccinia virus Ankara expressing MVA-HF glycoprotein signatures were compared to the previously described CDR3s of MV-specific hybridoma clones derived from an independent set of OmniRat™ immunized with whole MV antigens (22). The largest of the identified HF-associated clusters (244 members) matched three CDR3s of MV-specific hybridoma cells, suggesting that this cluster is an MV-H or F protein-induced CDR3 signature (Figure 6). Similarly, our TT-associated clusters were compared to known human TT-specific IgH sequences (12, 23–25). The CDR3s from the TT-associated *cluster 4* matched 12 published human CDR3s (Figure 7A). This OmniRat™ CDR3 signature as well as the human CDR3s consisted of 15-mer CDR3s following the same amino acid pattern. Both humans and rats elicited a conserved paratope defined by a static motif “+QWLV” (+ = R/F; the “+” stands for the positively charged amino acids R and F) at the center of the CDR3, flanked by variable positions that are connected to the torso of the CDR3 (Figure 7B). This indicates that similar key positions are used even across species. The sequence similarity between the human and rat CDR3s ranged from 67 to 87% resulting from different torso amino acid compositions at the positions flanking the conserved binding motif (Figures 7C,D). To compare the structures of these CDR3s from human and rat origin, we performed 3D-homology modeling on their Fab fragments. Four human antibodies with available heavy and light chain sequences (24) and four selected OmniRat™ heavy chain sequences paired with the human light chains were modeled with Rosetta Antibody. Within the OmniRat™-human chimeric Fab fragments, the CDR3s formed torso structures ranging from unconstrained amino acid formations over short beta-sheets to rigid beta-sheet hairpin constructs (Figure 7C). Like the rats, human CDR3s exposed the key binding residues at the very tip of the CDR3 loop by a rigid beta-sheet hairpin formation of the torso that protruded out of the IgH core structure (Figure 7D). Together our results corroborate the evolution of functionally convergent CDR3s in different individuals and by different vaccines delivering the same antigen. Also, this strongly indicates that OmniRat™ and humans, albeit the lower sequence similarity between their TT-associated CDR3s, produce antibodies with highly homologous CDR3s in response to the same antigen.

## Discussion

We analyzed more than 2,700,000 functional IgH sequences derived from the BM of transgenic rats expressing human B cell receptor genes immunized with different antigens. Our study showed that these rats produced identical as well as highly similar CDR3 amino acid sequences in response to common antigenic challenges. When shared CDR3 repertoire fractions were investigated at different levels of sequence similarity, overlaps between rats from the same vaccination group were optimal around 80% CDR3 amino acid similarity. Applying a differential gene expression workflow to the counts of 80% similar CDR3s, we presented a novel way to identify convergent, stereotypic CDR3 sequences in response to an antigenic stimulus. These included known CDR3s induced by different measles antigens, indicating that the identified CDR3s are specific for the MV H or F proteins which were shared in both immunizations. In addition, our approach also identified CDR3s in response to TT that were remarkably similar to known tetanus-specific CDR3s from human samples. Our findings highlighted the presence of convergent IgH transcripts at high levels in the BM of the transgenic rats and that these sequences are highly similar to those of humans.

Pairs of rats within the same group shared more identical CDR3s than pairs from different groups, but very few CDR3s were shared among all rats of a group. Given the tremendous size and diversity of the Ig repertoire, finding identical sequences in several individuals is indeed unlikely (7). Because of private processes during B cell development including stochastic affinity maturation of the Ig molecules, a certain variability in CDR3s converging toward reactivity with the same antigen is to be expected (6, 46, 47). Galson et al. found that for the identification of public repertoires in humans an 87.5–91.6% cutoff (1 in 12 to 1 in 8 amino acids) was optimal to identify TT- and influenza-related CDR3 clusters (48). In the present study, we explored the relation between CDR3 sequence similarity and the overlap between CDR3 repertoires, by inter- and intragroup cross-comparisons at different levels of sequence similarity. Our data showed that 80% amino acid similarity optimized the intragroup overlap between CDR3 repertoires while keeping the intergroup overlap at a minimum. The identified antigen-associated clusters were absent in rats outside immunization groups, which provides a strong support for their underlying biological relevance.

We showed that between 6 and 46% of the BM IgH repertoire correspond to convergent CDR3 sequences. Similar proportions (15–50%) of antigen-specific CDR3 sequences were reported in peripheral blood B cells of patients with acute dengue infections (5). In contrast, in the reconvalescent dengue patients as well as in influenza patients, convergent sequences represented only less than 1% of peripheral B cell sequences. Such human studies are normally restricted to peripheral blood where only a small fraction of the repertoire can be found and assessed (13, 49). Blood is the only readily available source for sampling B cells in humans. However, the high turnover rate of 5 × 10^11^ B cells per day (50) and low sampling depth make it difficult to capture a significant fraction for exhaustive analysis of antigen experienced Ig repertoires in a vaccination context (14, 51). In contrast, high levels of antigen-selected B cells can be found within the BM, where about 17% of all B cells reside, making this tissue a preferable target for studying the antigen-specific B cell response after vaccination. In this regard, elevated levels (37%) of public clones were observed in mice in response to HBsAg and less to NP-HEL (22%) and OVA (14%), when examining BM-derived long-lived plasma cells (CD138^+^ CD22^−^ MHCII^−^ CD19^−^ IgM^−^ PI^−^) (14). In the present study, we analyzed Ig mRNA from bulk rat BM cell isolates, an organ rich in serum antibody-producing plasma cells (15–17). Because plasma cells express significantly higher levels of Ig mRNA [estimated 500:5:2 compared to memory or naïve B cells (52)] and only class switched BCR (IgG) were targeted, those are overrepresented in our data sets (47, 48). This explains the high fractions of converging CDR3s we found in the assessed BM IgG repertoire. We thus primarily targeted antigen-associated effector B cells, facilitating the tracking of antigen-specific sequences induced by similar antigens.

The IgH repertoire of OmniRat™ displayed a CDR3 length distribution comparable to that of human IgG B cells isolated from peripheral blood and lower than that of the corresponding IgM B cells (34). This indicates that OmniRat™ is generating CDR3 diversity in an equivalent way as humans. Interestingly, the average mutation rate of OmniRat™ IgG sequences was much lower as human blood-derived IgG or IgM B cells. This is most likely due to the much younger Ig repertoire of the rats having not been exposed to a large number of antigens as compared to humans, leaving their repertoires relatively unchallenged and thus unmutated (14).

The clusters of antigen-induced CDR3s exhibited a diverse IgHV gene usage from mainly one predominant IgHV gene family. Only CDR3 *cluster 2* of the animals in the combined vaccination group MVA-HF + MVA exhibited significant differences in IgHV gene family usage across the rats. Similarly, different IgHV gene families were found in convergent CDR3 responses to Dengue infections and antipolysaccharide vaccination in humans implying a convergent evolution (5, 12). In an antigen–antibody binding scenario, the IgHV gene encodes for a structural scaffold while the CDR3 must precisely fit the antigen surface (3). Hoogenboom and Winter concluded from their synthetic antibody library that substitution of the CDR3 alone can create entirely different antibody specificities (53). However, in contrast to the other 29 clusters, the 15-amino acid long signature of MVA-HF + MVA cluster 2 was largely composed of the IgJH 6 gene segment resulting in a tyrosine-rich “YYYGMDV” tail motif, with a shorter section from the more diverse D/N region. Similarly, the signatures reported by Parameswaran and colleagues in response to Dengue were composed largely of a long, tyrosine-rich tail motif resulting from the IgJH region with very short D/N sections (5). We expect that such CDR3 signatures exhibit a rather polyreactive binding mediated by the abundant tyrosine residues. Yet, the absence of any 80% relatives in the other vaccination group underlines their presence as a result of the antigen exposure through the applied vaccination.

The varying IgHV gene assignments in each cluster can be explained by the relatively short read length of our approach. In this regard, IgHV genes belonging to the same family (e.g., IgHV4-39 and IgHV4-34) share a high level of similarity, only differing by few nucleotides. As the amplicons of the rat IgH transcripts did not span the whole IgHV sequence, information encoded in the FR1 and CDR1 are lost. This could easily lead to varying gene assignments, explaining the IgHV gene variability of the described CDR3 clusters.

Potential influence on the repertoire composition could result from PCR amplification biases introduced during library preparation as well as sequencing errors (54). We did not account for potential errors and sequencing bias by using molecular barcodes or similar methods (52, 55–57). However, the data analysis of the present study was based on collapsed, unique nucleotide Ig sequences, minimizing the influence of potential PCR amplification bias. Analysis of the nucleotide sequences before and after collapsing to unique nucleotide sequences revealed no major difference in our findings, indicating that PCR amplification bias did not falsify our results. The IonTorrent PGM sequencing platform is prone to insertion and deletion errors, especially within homopolymer repeats (58). Such errors cause frameshifts within the Ig sequence which are detected by IMGT with 98% efficiency in a benchmarking setup, missing only indels at the beginning and end of the sequence or if placed in close proximity to each other masking the resulting frameshift (59). Sequences with detected indels are marked by IMGT as productive with detected errors and were not included in the described analysis. Furthermore, our analysis is based on the CDR3 amino acid sequence. An insertion or deletion within the CDR3 encoding nucleotides results in the sequence being labeled as unproductive, with no correction attempts undertaken by IMGT (59). Less than 1% of indel combinations remains undetected by IMGT and could be present within the CDR3 encoding nucleotides (59). These rare combinations of sequencing errors would then result in artifactual CDR3s either covered by the applied 80% sequence similarity clustering threshold or missed because of higher sequence variation. Therefore, such CDR3 artifacts can be expected to induce only a small underrepresentation of CDR3s by lowering CDR3 counts. In conclusion, the presented workflow is well protected from potential sequencing errors or PCR bias that could impact our conclusion.

We found that certain CDR3s have high counts of 80% relatives within a group but very few to none in the unrelated groups. This is in principle comparable to differential gene expression in RNA-seq data. The CDR3 counts followed a negative binomial distribution but, unlike in RNA-seq experiments, our data contained large amounts of CDR3s with zero counts over different samples. These correspond to private CDR3s that are absent in other rats of the same or other groups. On the other hand, some CDR3s exhibited very high counts of 80% relatives within an animal. While such a data distribution is uncommon in RNA-seq, they were nevertheless compatible with our computational approach [DESeq2 (38)], as demonstrated by negative binomial data distribution and perfect sample grouping after VST of the CDR3 counts. Interestingly, the Euclidian distance grouping of MVA-HF and MVA rats remained unchanged for 85% and even for 75% CDR3 counts in contrast to TT-associated rats. Similarly, Trück et al. found highly similar (≤2 mismatches) Hib- and TT-related sequences enriched 7 days postvaccination, but could not identify H1N1- and MenC-related sequences at the same threshold (12). Correspondingly, statistical evidence of convergent CDR3s in pairs of donors against influenza with a mean genetic distance of ~75% was reported (60). Together with our data this indicated that the identification of convergent Ig repertoire responses using amino acid similarity thresholds was applicable. Future research will tell to what extent the 80% threshold can be applied to other antigens.

Identified convergent CDR3 matched to sequences of previously described human monoclonal antibodies against TT protein (12, 23–25). Despite the relatively low sequence similarity (67–87%) between OmniRat™ and human TT-specific CDR3s, they shared a common sequence and structural motif at the center of the CDR3. The center part of the CDR3 is exposed at the tip of the loop structure which directly interacts with the antigen while the adjacent amino acids act as a supporting scaffold. Similarly, Greiff and coworkers observed stereotypical motifs at the center of the CDR3 amino acid sequences in specific antibodies following NP vaccination in mice (14). While structural similarity cannot readily be used to determine antibody specificity, algorithms to identify convergent CDR3s could be further improved by including structural parameters drawn from the expanding amount of available crystal structures.

In conclusion, we demonstrated a strong public IgH response with converging and overlapping CDR3 repertoires in animals exposed to the same antigens. These converging repertoires consisted of similar CDR3 sequences that can be best described using an 80% amino acid similarity threshold. Additionally, we presented an approach to identify such CDR3s by adopting a group-wise expression analysis, similar to RNA-seq approaches. This provides also a valuable tool for large-scale HTS data mining to identify potential candidates for high-affinity targeted antibody design.

## Ethics Statement

This study was carried out in accordance with the recommendations of the Guide for the Care and Use of Laboratory Animals as approved by the Comité National d’Ethique de Recherche (CNER, Luxembourg).

## Author Contributions

J-PB and AD contributed equally to the work. J-PB designed and developed the bioinformatics approach, interpreted data, performed data processing, and wrote the manuscript. AD designed and carried out research, prepared samples, interpreted data, and wrote the manuscript. WF supported bioinformatics approaches and data processing and corrected the manuscript. AW-B set up the raw data processing script and performed data processing, data interpretation, and corrected the manuscript. SF and EC provided technical assistance with immunizations, ELISA, and virus culture. RS performed IonTorrent PGM sequencing. CM designed research, interpreted data, corrected the manuscript, and supervised work. All authors have read and approved the final version of the manuscript.

## Conflict of Interest Statement

The authors declare that the research was conducted in the absence of any commercial or financial relationships that could be construed as a potential conflict of interest.

## Footnotes

^1^www.imgt.org.

^2^https://www.ncbi.nlm.nih.gov/sra.

^3^www.graphpad.com.

^4^http://rosie.rosettacommons.org/.

^5^http://pymol.org.

## Abbreviations

ALUM, aluminum hydroxide; BaP, benzo[a]pyrene; BaP-TT, benzo[a]pyrene tetanus toxoid conjugate; BM, bone marrow; EPT, endpoint titers; F, fusion glycoprotein of the measles virus; FDR, false discovery rate; H, hemagglutinin glycoprotein of the measles virus; HF, hemagglutinin and fusion protein of the measles virus; HTS, high-throughput sequencing; IgL, immunoglobulin light chain; IMGT, ImMunoGeneTics database; MV, measles virus; MVA, Modified Vaccinia virus Ankara; ROSIE, Rosetta Online Server that Includes Everyone; TT, tetanus toxoid; VST, variance stabilizing transformation.
