# Supplementary material for: Functionally Convergent B Cell Receptor Sequences in Transgenic Rats Expressing a Human B Cell Repertoire in Response to Tetanus Toxoid and Measles Antigens
Source: Front Immunol. 2017 Dec 22;8:1834. doi: 10.3389/fimmu.2017.01834 (PMC5743747; doi:10.3389/fimmu.2017.01834)
Supplement: Supplementary file 1 [file Data_Sheet_1.PDF]

Supplemental Data

Table S1: OmniRat™ IgHV genes

IgHV gene family	IgHV gene
**IgHV1**	IGHV1-2
	IGHV1-24
	IGHV1-3
	IGHV1-8
**IgHV2**	IgHV2-26
	IgHV2-5
**IgHV3**	IgHV3-7
	IgHV3-9
	IgHV3-11
	IgHV3-22-2
	IgHV3-23
	IgHV3-30
	IgHV3-33
	IgHV3-35
	IgHV3-38
**IgHV4**	IgHV4-28
	IgHV4-31
	IgHV4-34
	IgHV4-39
	IgHV4-4
**IgHV6**	IgHV6-1
**IgHV7**	IgHV7-4

Table S2: High-throughput sequencing results

Vaccination	ID	Raw-reads w. MID	Filtered unique nt sequences	Unique CDR3s
**MVA-HF n=6**	1	1,365,752	75,632	4,007
	2	1,072,907	87,632	5,680
	3	1,573,425	97,070	5,050
	4	1,579,703	105,168	5,609
	5	1,110,541	53,913	3,536
	6	1,073,485	68,542	4,469
**MVA n=6**	7	929,621	75,643	4,333
	8	995,002	91,300	5,084
	9	937,392	65,840	3,288
	10	1,084,088	129,977	7,232
	11	998,174	105,000	4,980
	12	1,198,061	129,219	5,691
**BaP-TT n=5**	13	1,177,010	94,218	3,191
	14	990,929	91,500	5,838
	15	989,221	80,886	5,538
	16	1,126,831	89,527	4,399
	17	1,646,696	50,234	2,379
**TT n=4**	18	1,184,407	105,527	3,912
	19	1,119,688	65,630	4,228
	20	1,036,147	54,763	4,471
	21	1,380,809	137,371	6,756
**Alum n=6**	22	1,431,185	65,363	3,586
	23	1,879,372	85,192	7,202
	24	1,004,133	78,375	8,013
	25	896,017	68,058	6,602
	26	879,714	46,409	3,946
	27	850,298	86,623	4,365
**NEG n=5**	28	929,883	60,509	5,619
	29	1,121,113	100,287	5,885
	30	938,306	106,170	6,422
	31	1,380,088	135,281	6,809
	32	1,593,984	84,948	4,026
**Total**		37,473,982	2,771,807	162,146
**Mean± SD**		1,171,062 ±257,905	86,619 ±24,056	5,067 ±1,335

The first column indicates the vaccination group including the number of animals. The second column is the study-number of the animal. The third column shows the raw reads per animal as identified per MID. The fourth column shows the quality filtered reads, collapsed to unique nt sequences. The fifth column shows the number of unique CDR3 amino acid sequences after IMGT processing. The last two rows show the total, mean and standard deviation per column.

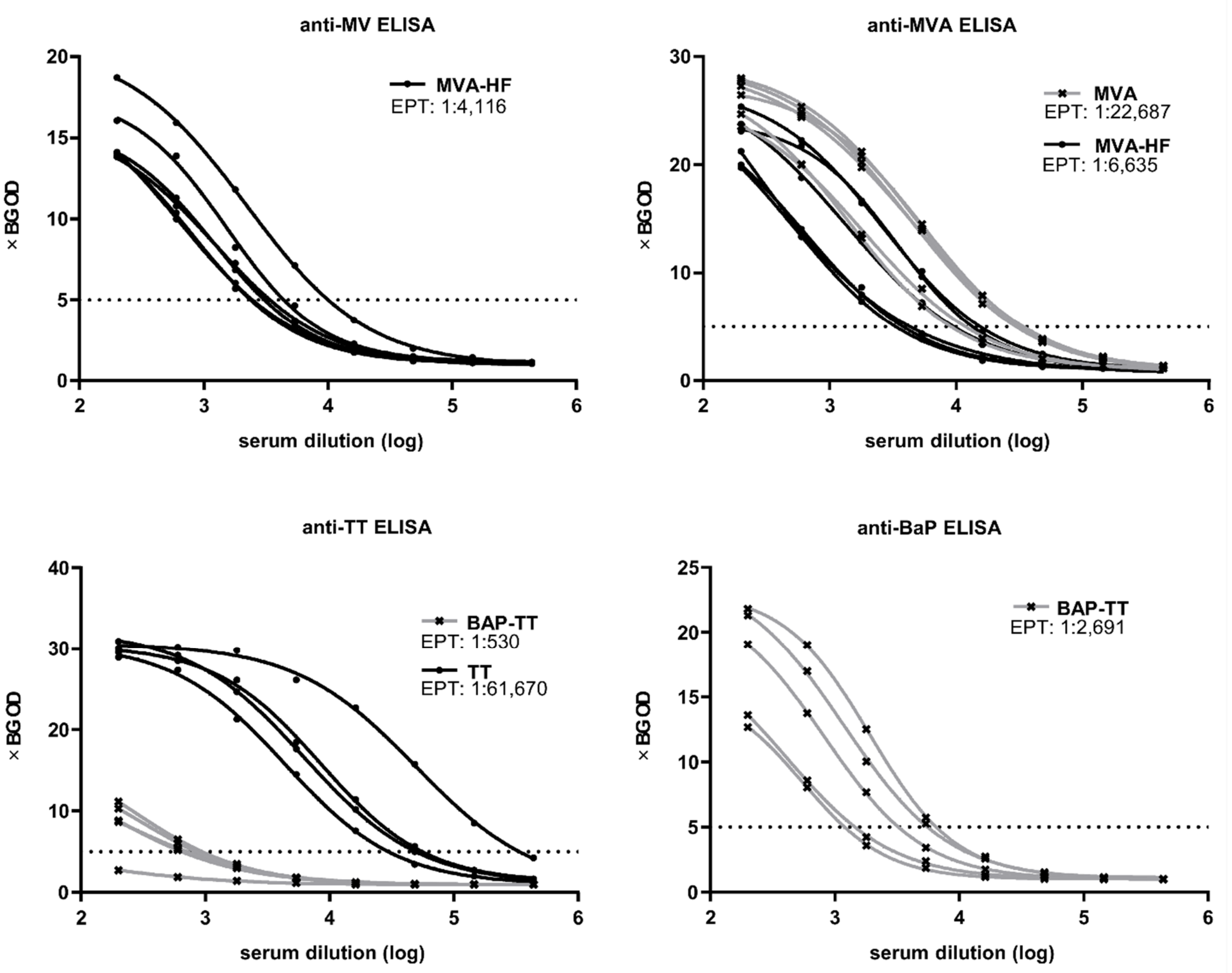

Figure S1. ELISAs. Antigen-specific serum IgG in immunized rats were measured by indirect ELISA. The serum dilution was plotted (log scale) against binding, measured by absorbance expressed as multiples of the average optical density of the background (BG OD). Average endpoint titers (EPT) were determined as the serum dilution corresponding to five times the BG OD (dotted line).

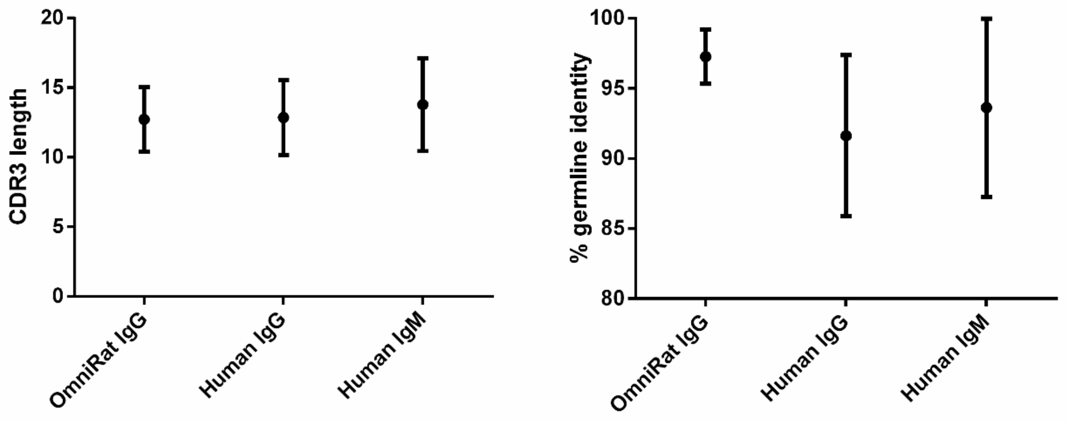

Figure S2. OmniRat™ repertoire characteristics compared to human. First panel shows CDR3 length for each sample as average with bars representing standard deviation. Second panel shows level of somatic hypermutation as percent of germline identity, determined by IMGT. Values are shown as average with error bars representing standard deviation.

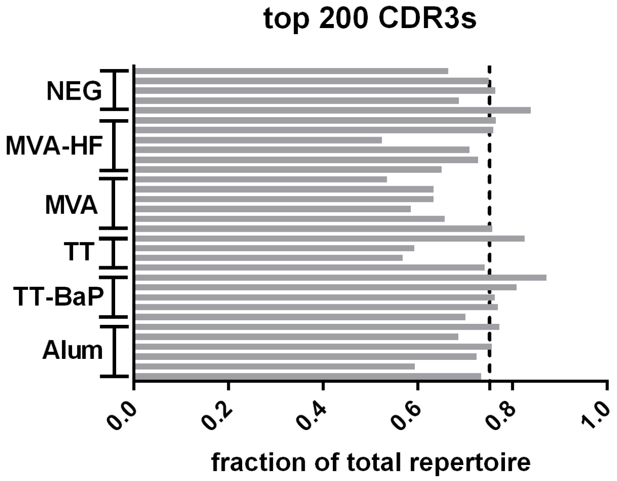

Figure S3. Top 200 CDR3 cutoffs. Bar chart displaying the repertoire size included in the top 200 CDR3s used for sample cross comparisons. Bars represent individual rats. Dotted line represents mean value across all samples.

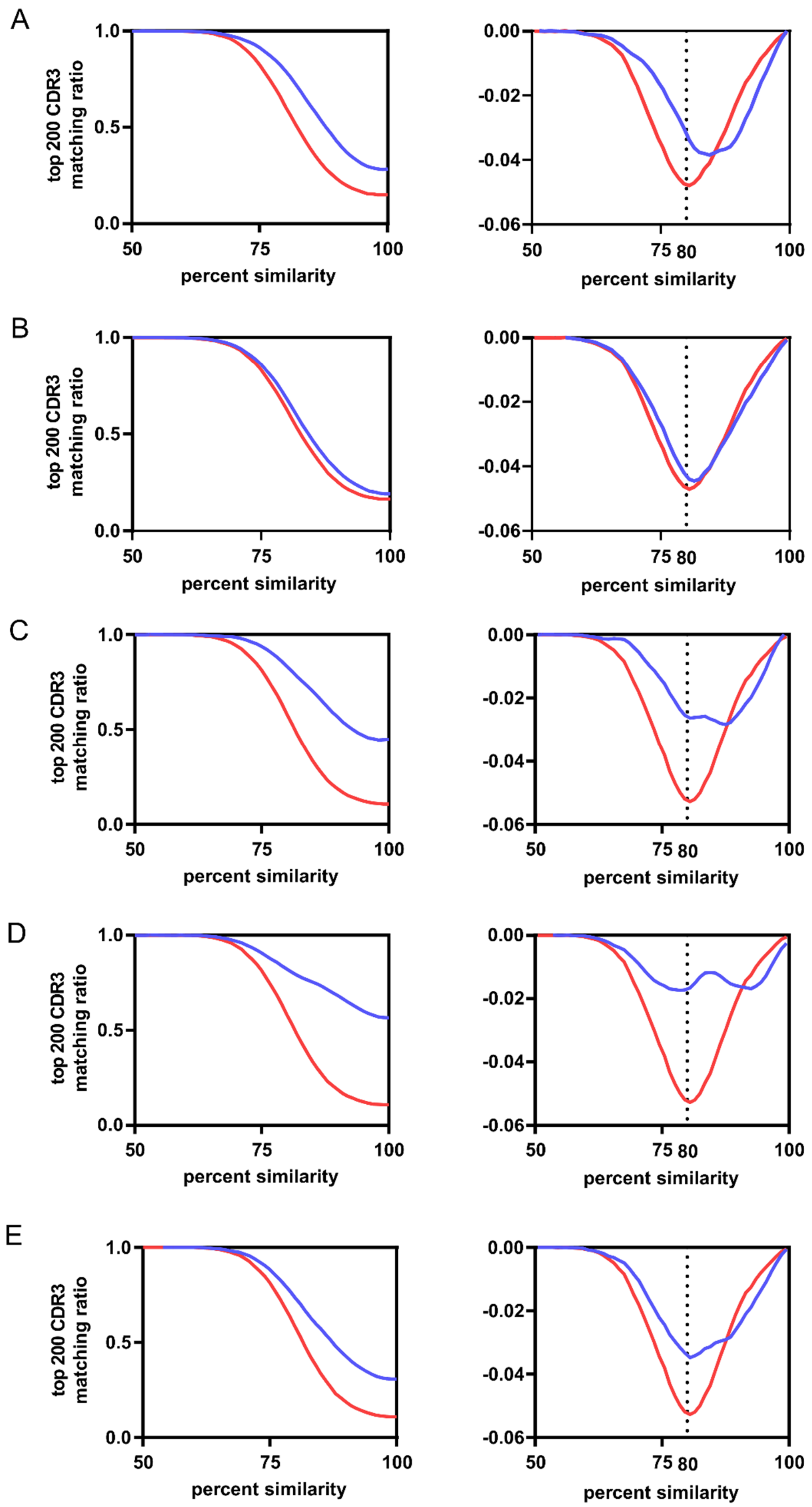
A
B
C
D
E
top 200 CDR3 matching ratio
1.0
0.5
0.0
0.00
-0.02
-0.04
-0.06
50
75
80
100
percent similarity

Figure S4. Influence of CDR3 sequence similarity on the CDR3 repertoire overlap between rats. (A) Left panel: Average fractions of top 200 CDR3s of the MVA vaccination group shared with all CDR3s of other samples. Samples were divided into two groups having either the same antigen (MVA-group samples, blue curve) or different antigens (ALUM, BaP-TT, TT and NEG samples, red curve). Both curves follow a similar sigmoidal behavior. Right panel: First derivative of both curves. Inflection points align at 80% CDR3 amino acid similarity. (B) shows the curves for the combined groups MVA-HF + MVA; (C) shows the curves for the BaP-TT vaccination group; (D) shows the curves for the TT vaccination group; (E) shows the curves for the combined groups BaP-TT + TT.

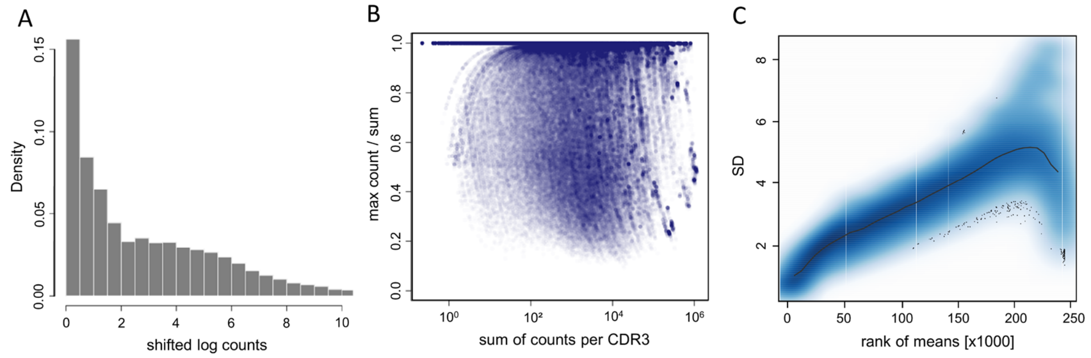

Figure S5. DESeq2 statistics. (A) Density-histogram representing the distribution of CDR3-counts (log(x+1) transformed). The CDR3-counts follow a negative binomial distribution (B) Sparsity-plot displaying the count distribution per CDR3. The sum of counts for every CDR3 is plotted in log10-scale against the highest count for the CDR3 divided by the sum of all counts for the CDR3. Density of data is indicated by hue. (C) CDR3-wise standard deviation of ranked means of counts after variance stabilizing transformation (VST-counts). The black line shows the standard deviation for all ranked means of VST-counts across all samples, the blue area indicates the data distribution and density by hue.

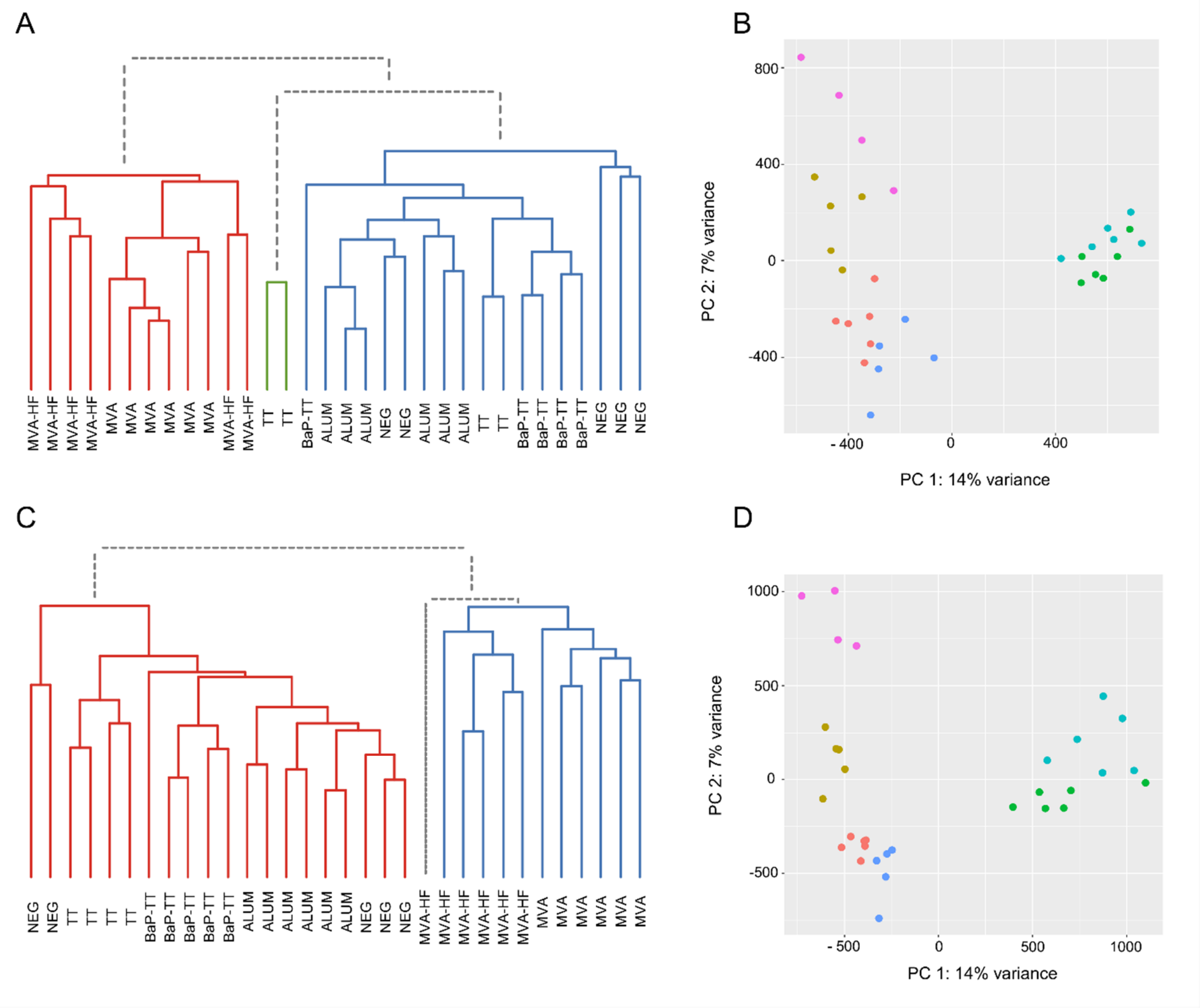

Figure S6. Sample grouping for 75 and 85% CDR3 similarity counts. (A) Dendrogram of the Euclidian sample distances calculated for VST-counts based on 75% CDR3 similarity. Three main clusters are indicated by coloration (Cluster I: red, Cluster II: green, Cluster III: blue). (B) Scatterplot for the first two principal components of VST-counts based on 75% CDR3 similarity. Samples are colored by vaccination-group (MVA: light blue, MVA-HF: green, TT: pink, BaP-TT: gold, ALUM: red, NEG: blue). (C) Dendrogram of the Euclidian sample distances calculated for VST-counts based on 85% CDR3 similarity. Three main clusters are indicated by coloration (Cluster I: red, Cluster II: green, Cluster III: blue). (D) Scatterplot for the first two principal components of VST-counts based on 85% CDR3 similarity. Samples are colored like in (B).

Table S3: IgHV gene usage of overrepresented CDR3 clusters as average percentage over all rats and standard deviation.

		V1-24		V1-2		V1-3		V1-8		V3-11		V3-23		V3-30		V3-33		V3-7		V3-9		V4-28		V4-31		V4-34		V4-39		V4-4		V6-1		V7-4	
**BaP-TT**	1	-	-	0.19%	±0.13	-	-	0.04%	±0.01	0.09%	±0.07	0.22%	±0.00	-	-	-	-	0.02%	±0.00	0.01%	±0.00	1.79%	±2.45	3.72%	±1.79	10.89%	±4.11	78.33%	±12.15	7.57%	±3.70	1.44%	±0.94	0.04%	±0.02
	2	0.32%	±0.07	-	-	-	-	0.02%	±0.00	0.26%	±0.16	0.08%	±0.00	-	-	-	-	0.04%	±0.00	-	-	0.29%	±0.00	3.85%	±0.00	13.52%	±3.03	77.43%	±5.35	10.43%	±0.00	2.22%	±0.00	-	-
	3	-	-	-	-	-	-	-	-	-	-	-	-	-	-	-	-	-	-	-	-	7.14%	±0.00	-	-	6.90%	±0.24	85.95%	±7.38	7.14%	±0.00	-	-	-	-
	4	1.35%	±0.00	-	-	-	-	0.90%	±0.00	-	-	0.45%	±0.00	-	-	-	-	0.11%	±0.00	-	-	0.11%	±0.00	6.40%	±0.00	19.33%	±0.00	75.94%	±10.21	13.57%	±8.51	2.47%	±0.00	0.22%	±0.00
	5	-	-	-	-	-	-	-	-	-	-	-	-	-	-	-	-	-	-	-	-	-	-	9.09%	±0.00	10.55%	±0.00	71.56%	±2.49	4.55%	±0.00	4.24%	±1.51	-	-
**TT**	1	91.79%	±5.55	-	-	-	-	6.49%	±1.01	0.30%	±0.00	0.10%	±0.00	-	-	-	-	4.00%	±3.70	0.40%	±0.20	-	-	0.20%	±0.00	0.80%	±0.39	0.75%	±0.44	0.20%	±0.00	0.60%	±0.00	0.10%	±0.00
	2	1.27%	±0.55	-	-	-	-	0.42%	±0.00	-	-	-	-	-	-	-	-	0.88%	±0.17	-	-	1.05%	±0.00	2.58%	±1.64	24.62%	±4.44	61.41%	±4.14	6.45%	±3.05	3.05%	±0.40	1.82%	±0.00
	3	1.72%	±1.42	-	-	-	-	0.62%	±0.00	-	-	0.75%	±0.16	-	-	-	-	0.31%	±0.00	-	-	1.56%	±0.60	4.88%	±2.65	64.06%	±6.84	14.86%	±4.79	5.21%	±2.23	6.09%	±1.15	1.21%	±0.62
	4	0.95%	±0.46	-	-	-	-	0.50%	±0.00	3.36%	±0.87	93.10%	±6.95	0.70%	±0.00	1.35%	±0.64	2.40%	±0.41	3.05%	±0.93	-	-	0.60%	±0.10	0.50%	±0.00	1.49%	±0.00	-	-	1.00%	±0.00	-	-
	5	4.13%	±1.84	-	-	-	-	1.10%	±0.66	-	-	-	-	-	-	0.33%	±0.00	-	-	1.18%	±0.00	0.46%	±0.13	4.34%	±0.87	27.56%	±4.11	49.49%	±11.44	5.35%	±1.89	7.51%	±6.49	0.22%	±0.00
**BaP-TT + TT**	1	0.47%	±0.67	-	-	-	-	0.23%	±0.16	2.80%	±2.11	12.64%	±11.63	0.37%	±0.22	0.75%	±0.60	4.38%	±3.96	78.42%	±12.60	0.03%	±0.01	0.06%	±0.02	0.09%	±0.04	0.07%	±0.03	0.06%	±0.07	0.62%	±0.61	0.05%	±0.03
	2	3.00%	±2.23	-	-	-	-	1.90%	±2.65	62.76%	±16.58	21.93%	±9.16	0.29%	±0.20	1.92%	±1.35	8.33%	±2.67	7.72%	±3.33	0.04%	±0.01	0.27%	±0.11	0.84%	±0.43	2.46%	±2.72	0.48%	±0.19	1.93%	±2.02	0.21%	±0.13
	3	0.75%	±0.64	-	-	-	-	0.11%	±0.00	2.58%	±1.58	49.36%	±39.98	1.00%	±0.67	1.44%	±1.01	5.34%	±2.93	72.09%	±31.19	-	-	0.11%	±0.00	-	-	3.33%	±0.00	0.11%	±0.00	3.53%	±3.70	-	-
	4	-	-	0.64%	±0.00	-	-	0.22%	±0.00	2.73%	±1.65	54.34%	±45.66	-	-	0.38%	±0.06	4.71%	±3.62	87.64%	±10.25	-	-	0.44%	±0.00	0.11%	±0.00	0.43%	±0.00	0.21%	±0.00	1.07%	±0.00	0.27%	±0.05
	5	-	-	1.29%	±0.40	-	-	0.45%	±0.17	1.25%	±0.45	93.65%	7.24	0.46%	±0.10	1.45%	±0.60	4.03%	±1.21	2.37%	±0.50	-	-	0.23%	±0.05	0.53%	±0.00	1.16%	±0.54	0.09%	±0.00	0.50%	±0.21	2.40%	±0.00
**MVA-HF**	1	1.14%	±0.69	-	-	-	-	0.29%	±0.15	0.05%	±0.00	0.27%	±0.01	-	-	0.21%	±0.21	0.11%	±0.04	0.28%	±0.29	0.06%	±0.01	6.07%	±3.13	20.11%	±4.11	66.30%	±5.68	2.00%	±0.78	3.19%	±1.75	1.55%	±1.35
	2	-	-	-	-	-	-	1.32%	±0.91	4.60%	±0.16	11.35%	±6.43	0.16%	±0.00	2.22%	±0.00	4.89%	±0.44	84.41%	±12.70	-	-	0.08%	±0.00	-	-	-	-	-	-	-	-	-	-
	3	1.39%	±0.00	-	-	-	-	-	-	-	-	0.08%	±0.00	-	-	-	-	0.24%	±0.00	0.46%	±0.00	0.08%	±0.00	0.58%	±0.34	1.14%	±0.68	1.50%	±0.00	0.08%	±0.00	97.58%	±1.74	-	-
	4	-	-	-	-	-	-	-	-	-	-	-	-	-	-	-	-	-	-	-	-	-	-	9.09%	±0.00	28.81%	±20.24	74.98%	±21.78	-	-	4.55%	±0.00	-	-
	5	-	-	87.73%	±1.73	-	-	3.55%	±0.20	-	-	9.21%	±1.02	-	-	-	-	-	-	-	-	-	-	-	-	-	-	-	-	-	-	-	-	-	-
**MVA**	1	-	-	0.53%	±0.49	0.01%	0.00%	0.09%	±0.03	0.05%	±0.03	0.32%	±0.08	0.02%	±0.00	0.09%	±0.05	0.11%	±0.02	0.09%	±0.03	0.07%	±0.03	0.80%	±0.24	1.72%	±0.78	2.19%	±1.03	0.33%	±0.11	93.88%	±1.98	0.13%	±0.07
	2	-	-	0.23%	±0.10	-	-	0.08%	±0.00	0.33%	±0.00	0.27%	±0.02	0.03%	±0.00	0.29%	±0.30	0.09%	±0.07	0.22%	±0.11	0.08%	±0.00	1.00%	±0.30	3.80%	±3.18	3.93%	±3.05	0.21%	±0.13	93.74%	±6.32	0.39%	±0.26
	3	95.09%	±4.55	-	-	-	-	3.55%	±0.86	0.49%	±0.30	1.79%	±1.25	-	-	0.15%	±0.00	0.47%	±0.33	0.14%	±0.00	-	-	-	-	0.42%	±0.37	0.24%	±0.09	0.87%	±0.72	0.19%	±0.05	1.36%	±1.29
	4	0.07%	±0.00	-	-	-	-	-	-	-	-	0.20%	±0.00	0.07%	±0.00	0.20%	±0.00	0.07%	±0.00	-	-	0.07%	±0.00	0.57%	±0.15	1.27%	±0.22	0.74%	±0.11	0.26%	±0.00	97.88%	±1.58	0.26%	±0.00
	5	3.07%	±1.52	-	-	-	-	1.73%	±1.04	55.87%	±8.14	25.12%	±8.67	0.68%	±0.00	5.08%	±1.99	3.21%	±0.15	4.50%	±1.07	0.14%	±0.00	1.11%	±0.43	1.37%	±0.00	2.39%	±1.53	0.27%	±0.00	1.74%	±1.33	1.63%	±0.11
**MVA-HF + MVA**	1	1.04%	±0.52	-	-	-	-	0.42%	±0.20	2.19%	±0.90	88.08%	±4.08	0.25%	±0.16	2.67%	±1.29	1.31%	±0.97	2.43%	±0.87	0.03%	±0.03	0.22%	±0.15	0.27%	±0.21	0.31%	±0.19	0.13%	±0.11	0.35%	±0.18	0.32%	±0.27
	2	14.66%	±12.54	-	-	1.52%	1.84%	1.74%	±1.82	3.24%	±3.75	5.22%	±8.56	0.29%	±0.43	1.46%	±3.15	3.61%	±7.26	1.77%	±2.29	0.47%	±0.32	4.99%	±5.98	18.21%	±10.50	27.40%	±11.58	2.62%	±1.91	12.70%	±12.58	0.79%	±0.40
	3	0.44%	±0.43	-	-	0.01%	0.00%	0.19%	±0.22	0.11%	±0.12	0.36%	±0.26	0.03%	±0.01	0.10%	±0.10	0.22%	±0.24	0.22%	±0.27	0.09%	±0.11	0.49%	±0.15	1.16%	±0.41	1.66%	±0.82	0.27%	±0.19	95.93%	±2.63	0.23%	±0.25
	4	-	-	2.39%	±2.72	-	-	0.61%	±0.51	3.15%	±2.52	83.58%	±8.67	0.16%	±0.08	3.52%	±2.40	1.21%	±0.44	3.37%	±2.43	0.04%	±0.03	0.30%	±0.16	0.36%	±0.22	0.50%	±0.25	0.23%	±0.20	0.55%	±0.19	0.46%	±0.74
	5	1.04%	±1.51	-	-	-	-	1.88%	±2.34	0.12%	±0.07	0.43%	±0.57	0.16%	±0.00	0.04%	±0.03	0.10%	±0.06	0.08%	±0.04	0.08%	±0.06	5.32%	±8.55	4.30%	±4.97	5.18%	±8.43	6.29%	±13.45	84.23%	±22.04	0.27%	±0.16
